# Genetic control of wheat flour end‐use quality and rheology by genome‐wide association studies

**DOI:** 10.1002/tpg2.70236

**Published:** 2026-04-16

**Authors:** Juan Menor de Gaspar, Richard D. Cuthbert, Ruan Yuefeng, Ron Knox, BinXiao Fu, Kun Wang, Jatinder S. Sangah, Samia Berraies, Brad Meyer, Firdissa E. Bokore, Julio Isidro y Sánchez

**Affiliations:** ^1^ Centro de Biotecnologia y Genómica de Plantas (CBGP, UPM‐INIA), Universidad Politécnica de Madrid (UPM)–Instituto Nacional de Investigación y Tecnologia Agraria y Alimentaria (INIA) Campus de Montegancedo‐UPM Madrid Spain; ^2^ Agriculture and Agri‐Food Canada Swift Current Research and Development Centre Swift Current Canada; ^3^ Grain Research Laboratory Canadian Grain Commission Winnipeg Canada

## Abstract

Improving end‐use quality in bread wheat (*Triticum aestivum*) requires dissecting the genetic basis of complex processing traits and deploying robust prediction pipelines in breeding. We performed genome‐wide association studies (GWASs) using 1767 high‐quality single‐nucleotide polymorphisms generated by genotyping‐by‐sequencing in a diverse Canada Western Red Spring panel phenotyped near Swift Current, SK, from 2009 to 2019 for grain protein content, milling yield, mixing energy, water absorption, and dough extensibility.

The analysis detected significant marker–trait associations on 13 chromosomes, recovering signals at *Rht‐B1* and *Glu‐1* and revealing multiple additional signals that may represent previously unreported loci in this germplasm and environmental context, consistent with polygenic control. We then evaluated genomic selection using GBLUP (Genomic Best Linear Unbiased Predictor) and BayesB with and without including significant GWAS hits as fixed effects; gains in predictive accuracy were generally negligible, although water absorption showed modest improvement, compatible with fewer, larger effect loci. Functional annotation of genes near associated variants implicated stress responses, protein metabolism, and grain filling.

Together, these results refine the genetic architecture of Canadian wheat quality and support integrating GWAS‐informed biology with genome‐wide prediction to accelerate quality‐by‐design breeding.

## Plain Language Summary

Wheat is a major food source worldwide, and improving its quality is essential for better bread and flour production. This study used genetic analysis to find DNA markers linked to important wheat quality traits like protein content, milling yield, and dough properties. We analyzed a large dataset from Canadian wheat varieties and identified 29 significant genetic regions affecting these traits. Some were near known genes, while others were new discoveries. We also tested whether using these genetic markers in prediction models could improve wheat breeding. The results showed only slight accuracy gains, except for water absorption. Our findings provide valuable genetic insights that can help breeders develop high‐quality wheat varieties more efficiently. This research supports the use of advanced genetics to improve food quality and production.

**Abbreviations**
BU: Brabender units
GPC: grain protein content
GS: genomic selection
GWAS: genome‐wide association study
LOD: logarithm of the odds
PCs: principal components
QTL: quantitative trait loci
SCRDC: Swift Current Research and Development Center
SNP: single nucleotide polymorphism

## 1 INTRODUCTION

The global demand for staple crops has increased in recent decades due to population growth and evolving consumer preferences (Ray et al., 2013). Wheat (*Triticum aestivum* L.) is among the most widely cultivated cereals and is a major source of calories and protein worldwide (Erenstein et al., 2022; FAO et al., 2023). Consequently, improving grain yield and end‐use quality traits in cereal crops, particularly wheat, has become a primary objective for breeders, agronomists, and policymakers. Bridging these two crucial goals is not without its challenges. In practice, increasing yield can sometimes come at the expense of grain quality, as the genetic and physiological trade‐offs complicate simultaneous improvements. Environmental variability and resource constraints further intertwine yield and quality responses (DePauw et al., 2007). Maintaining key quality attributes, such as grain protein content (GPC) and processing performance, requires understanding complex genetic architectures and genotype‐by‐environment interactions (Michel et al., 2019; Simmonds, 1995). Advances in quantitative genetics, genome‐wide association studies (GWAS), and genomic selection (GS) provide a framework to dissect these traits and accelerate improvement.

Quality in bread wheat spans compositional, functional, and rheological dimensions. Milling yield, typically quantified with experimental mills that approximate commercial streams, estimates flour extraction from whole grain (Campbell et al., 2001; Finney & Andrews, 1986; McCartney et al., 2006). Dough mixing behavior, routinely assessed with mixographs or farinographs, captures attributes such as peak time, peak height, and dough tolerance, which reflect gluten strength and elasticity (Bloksma et al., 1988; Campbell et al., 2001; McCartney et al., 2006; X. Huang et al., 2006). Water absorption, measured via farinograph or alkaline water retention capacity, gauges the flour's capacity to absorb and retain moisture and relates to GPC and starch damage (Campbell et al., 2001; Miller et al., 1997; Yamazaki, 1953). Extensograph profiles add information on dough strength, extensibility, and fermentation behavior, linking laboratory metrics to baking performance (Bangur et al., 1997). However, extensograph‐based phenotyping is resource‐intensive: it requires large flour quantities, precise milling, and long rest times after determining farinograph absorption, which limits throughput for large breeding populations. Labor and time‐intensive protocols (Bass & Pomeranz, 1988) can slow genetic gains, although high‐throughput phenotyping is beginning to ease these constraints (Fu, Wang, Dupuis, & Cuthbert, 2017).

Historically, quantitative trait loci (QTL) mapping provided the first insights into genetic control of quality traits (Campbell et al., 2001; Zanetti et al., 2001), but mapping resolution and allelic diversity were often limited. With declining genotyping costs, GWAS emerged as a powerful approach to detect marker–trait associations (MTAs) across diverse germplasm with higher mapping precision (Guo et al., 2023; White et al., 2022). Numerous GWAS and QTL mapping studies have successfully dissected wheat's genetic variation for abiotic and biotic stress tolerance (Devate et al., 2022; Saini et al., 2021) and a range of agronomic traits across diverse environments (Pang et al., 2021; P. Hu et al., 2021). Nevertheless, for expensive, low‐throughput end‐use traits, particularly those requiring extensograph measurements, GWAS studies remain scarce (Bordes et al., 2011; Jin et al., 2023; Zhao et al., 2024). This limited focus reflects the logistical constraints associated with sample size, labor‐intensive milling, and specialized instrumentation.

Known loci such as *Rht‐B1* and *Gpc‐B1*, associated with yield and GPC, underscore the importance of investigating interactions between novel and established genes to enhance flour performance (Flintham et al., 1997; Joppa et al., 1997). Gluten proteins, primarily glutenin and gliadin, are central to wheat's end‐use quality. High‐molecular‐weight glutenin subunits (HMW‐GS) contribute to dough elasticity and strength, while low‐molecular‐weight glutenin subunits and gliadins influence extensibility and viscosity (Campbell et al., 2001; D. Liu et al., 2023; Shewry et al., 2003; Sun et al., 2008; White et al., 2022). Variations in these subunits significantly affect rheological traits such as mixing energy, extensibility, and final loaf volume (Huebner & Wall, 1976; Nelson et al., 2006; Sharma et al., 2020; Zanetti et al., 2001; Zhou et al., 2021). To identify additional genes regulating quality traits, gene ontology (GO) analyses provide valuable insights by linking significant MTAs to biological processes, molecular functions, and cellular components (Bastos et al., 2011; Poretsky et al., 2024). Similar approaches in other crops, such as grape (García‐Abadillo et al., 2024), and even human health studies (Lebrec et al., 2009), underscore GO's utility in dissecting the genetic basis of complex traits.

GS complements locus discovery by capturing genome‐wide small effects to predict breeding values (Crossa et al., 2017). GS is especially attractive for costly traits where phenotyping is a bottleneck, such as extensibility and resistance to stretching (hereafter $R_{\max}$) (Subedi et al., 2023). Statistical frameworks range from linear mixed models (e.g., GBLUP) to variable selection methods (e.g., BayesB) that can perform well when major QTL contribute appreciably to trait variance (C. Wang et al., 2012). Integrating GWAS signals into GS by up‐weighting or fixing key markers may improve accuracy for traits with oligogenic components (Zhang et al., 2023), whereas fully polygenic traits often favor uniform shrinkage across the genome.

Here, we investigate the genetic architecture of high‐cost, end‐use quality traits in bread wheat using an integrated GWAS–GS framework. We target GPC, milling yield, mixing energy, water absorption, dough extensibility, and $R_{\max}$, pairing marker discovery with functional annotation to highlight candidate genes and biological pathways. We then benchmark GS accuracy for these traits and test whether incorporating GWAS‐identified loci improves prediction compared with standard genome‐wide models. Our results clarify the balance between polygenic background and larger‐effect contributors across traits and provide actionable guidance for deploying GWAS‐informed GS to accelerate quality‐by‐design breeding in Canadian wheat.

## 2 MATERIALS AND METHODS

### 2.1 Plant materials

We analyzed wheat grain samples from the Agriculture and Agri‐Food Canada Swift Current Research and Development Centre (SCRDC, Swift Current, SK, Canada) for end‐use quality at the Canadian Grain Commission's Grain Research Laboratory (Table 1). Except for grain protein concentration (GPC), we sourced grain for all traits from yield trials near Swift Current, SK. We measured GPC at 40 sites spanning multiple locations in western Canada from 2009 to 2019.

**TABLE 1:** Scope and summary of end‐use quality traits analyzed in the genome‐wide association study.

			Number of observations					Number of lines					Statistics				
**Trait**	Number of locations	Number of years	2009–2015	2016	2017	2018	2019	2009–2015	2016	2017	2018	2019	Max	Min	Mean	Sd	$h^{2}$
Extensibility	1	2	0	115	0	195	0	0	115	0	195	0	23.55	15.20	19.11	1.49	0.410
GPC	40	8	354	436	697	3330	763	137	76	225	982	191	18.4	8.60	13.70	1.69	0.941
Milling yield	1	2	0	115	0	195	0	0	115	0	195	0	79.09	72.08	75.59	1.34	0.698
Mixing energy	1	1	0	0	0	195	0	0	0	0	195	0	20.75	6.26	10.37	2.27	0.400
$R_{\max}$	1	1	0	0	0	195	0	0	0	0	195	0	936.5	226	461.07	132.43	0.735
Predicted water absorption	1	2	0	115	0	195	0	0	115	0	195	0	73.16	55.79	64.93	2.82	0.589

*Note*: We report, for each trait, the number of test locations and years, the number of plot‐level observations and unique lines evaluated in each period (2009–2015, 2016, 2017, 2018, and 2019), and descriptive statistics (Max, Min, Mean, and Sd) computed across all observations, together with narrow‐sense heritability ($h^{2}$) on an entry‐mean basis. Trait units: grain protein concentration (GPC, %), milling yield (%), predicted water absorption (%), extensibility (cm), mixing energy (W · h kg^−1^), and resistance to stretching ($R_{\max}$, Brabender units, BU). Zeros indicate that we did not measure the trait in that period.

We assembled several cohorts. First, we evaluated a doubled haploid (DH) population (B1292&) derived from *Carberry* × *Vesper* (111 progeny lines) together with checks (BW961, *Faller*) and the two parents: in 2016 for water absorption, extensibility, and milling yield; and for GPC in 2015 (one location) and 2017 (two locations). Second, we evaluated 195 entries comprising 10 F_4_ families from each of the 19 SCRDC hard red spring (HRS) wheat populations (B1606‐24GS), their parents, and check cultivars in 2018 for water absorption, extensibility, resistance to stretching ($R_{\max}$), mixing energy, and milling yield, and at four locations in 2018 for GPC. Third, we evaluated 191 entries comprising ten F_4_ families from each of 18 SCRDC HRS populations (2019 cycle), their parents, and checks for GPC at four locations in 2019. We further compiled GPC data from inbred lines in advanced and registration trials (A‐ and C‐trials; including Western Bread Wheat, Parkland Wheat, Canada Northern Hard Red, High Yield, and Central Bread Wheat trials). We also included 100 randomly selected DH lines from *Carberry* × *AC Cadillac* (B0767&) and all F_4_ lines from the 2018 HRS breeding program.

To clarify which breeding cohorts contributed to each phenotypic dataset we summarize cohort‐by‐trait coverage in Table 2. Briefly, all non‐GPC end‐use quality traits were evaluated only in the B1292& and B1606‐24GS cohorts, whereas the GPC dataset additionally included multiple cohorts from advanced and registration trials as well as the 2019 breeding cycle, forming a broader diversity panel.

**TABLE 2:** Cohort contribution to each phenotypic dataset.

Cohort / trial set	GPC	Predicted water absorption	Extensibility	Milling yield	Mixing energy	$R_{\max}$
B1292& (Carberry × Vesper DH + checks)	✓	✓	✓	✓	✗	✗
B1606‐24GS (2018 HRS F_4_ families + parents/checks)	✓	✓	✓	✓	✓	✓
Other cohorts (advanced/registration trials, 2019 cycle, etc.)	✓	✗	✗	✗	✗	✗

*Note*: Individuals from the B1292& and B1606‐24GS cohorts are highlighted in the principal component analysis (PCA) plot shown in Figure 1.

Checkmarks indicate that the cohort contributed observations for the corresponding trait in the trait in the genome‐wide association study (GWAS). Crosses indicate that the trait was not evaluated for that cohort. Abbreviations: GPC, grain protein concentration; $R_{\max}$, resistance to stretching.

When available, we composited grain samples from two replicates per line for quality analyses (instrumental replicates). We measured extensibility, milling yield, and water absorption on 310 unique lines and check cultivars across 2 years; we measured mixing energy and $R_{\max}$ on 195 lines and checks. The GPC data set comprised 1454 unique lines and checks, each with a variable number of observations, totaling 5580 plot‐level records.

We quantified GPC by calibrated near‐infrared reflectance spectroscopy and expressed it as a percentage (Owens et al., 2009). We measured the remaining quality traits following Fu, Wang, Dupuis, and Cuthbert (2017). Briefly, we milled grain with a modified Quadrumat Junior and expressed milling yield as a percentage. We predicted water absorption using a GlutoPeak device (Fu, Wang, & Dupuis, 2017) and expressed it as a percentage. We used the GlutoPeak‐inferred optimal hydration to mix 200 g of flour in a pin mixer and recorded mixing energy (W · h kg^−1^). After resting, we stretched dough on an extensograph to determine extensibility (cm) and $R_{\max}$ (Brabender units [BU]). All phenotypic measurements were obtained in the same laboratory using consistent, standardized protocols across years; for GPC, which was evaluated across multiple years and locations, environmental sources of variation were explicitly accounted for in the statistical models. For reference, phenotypic distributions of the analyzed traits are shown in Figure S1.

### 2.2 Genotyping

We generated genotypes using the *LGC Genomics* SeqSNP targeted genotyping‐by‐sequencing platform. We targeted a custom panel of 4520 markers from the Illumina Infinium 90K Wheat iSelect single nucleotide polymorphism (SNP) array (S. Wang et al., 2014), selected for informativeness in Canadian germplasm and spaced across the genome.

We processed genotype data in R using snpReady (Granato & Fritsche‐Neto, 2018; Team, 2024). We applied marker‐ and sample‐level quality control by removing non‐biallelic SNPs, excluding SNPs with minor allele frequency <0.05, and filtering out SNPs with more than 95% missing genotype calls. We also removed individuals with more than 95% missing genotypes. Missing genotypes remaining after filtering were imputed using Wright's method as implemented in snpReady (Granato & Fritsche‐Neto, 2018). Briefly, for a missing genotype $x_{ij}$ (individual i, marker j), Wright's method computes genotype probabilities as a function of the marker allele frequency $p_{j}$ and an individual‐specific inbreeding coefficient $F_{i}$ (estimated from the marker data), and imputes the missing call based on these probabilities (Equation 1). We selected this approach because it incorporates both marker‐ and individual‐level information (in contrast to mean imputation), minimizing imputation‐driven artifacts and remaining computationally efficient. After filtering and imputation, 1767 SNPs remained. Genotypes were coded as minor‐allele dosages {0,1,2} for downstream analyses.

(1) $$p\left(x_{ij}\right)=\left\{\begin{array}{cc} 0= & {\left(1-p_{j}\right)}^{2}+p_{j}\left(1-p_{j}\right)F_{i},\\ 1= & 2p_{j}\left(1-p_{j}\right)-2p_{j}\left(1-p_{j}\right)F_{i},\\ 2= & p_{j}^{2}+p_{j}\left(1-p_{j}\right)F_{i}, \end{array}\right.$$

### 2.3 Genome‐wide association study

We performed GWAS in GAPIT using the Bayesian‐information and linkage‐disequilibrium iteratively nested keyway (BLINK) method (M. Huang et al., 2018; J. Wang, 2024), which has been shown to provide increased statistical power and improved control of false positives compared to other GWAS approaches, while accounting for population structure and relatedness and remaining computationally efficient. To account for structure and relatedness, we included a centered kinship matrix and the first three principal components (PCs) (Larsson et al., 2013). To control type I errors, *p*‐values were adjusted using the Bonferroni correction with α=0.05. We generated Manhattan and *Q*–*Q* plots with CMplot (Yin et al., 2021). For traits measured once per genotype (all except GPC), we analyzed phenotypic values directly, as adjusting for year effects was not appropriate given the lack of replication and the complete absence of genotype overlap across years, which would confound year and genotype effects. For GPC, we first estimated genotype best linear unbiased estimates (BLUEs) from a mixed model (Equation 2) fitted to location‐level observations using *lme4* (Bates et al., 2015). With $y_{its}$ denoting the GPC observation of genotype i in year t, and location s. We fitted:

(2) $$y_{itsp}=\mu +g_{i}+u_{ts}+\varepsilon_{its},$$

where μ is the overall mean, $g_{i}$ is the fixed effect of genotype (BLUEs), and $u_{ts}$ is a random environmental effect defined as the year‐by‐location combination (i.e., locations nested within years), with $u_{ts}∼N\left(0,\sigma_{\mathrm{env}}^{2}\right)$. Residuals were assumed $\varepsilon_{its}∼N\left(0,\sigma_{\varepsilon }^{2}\right)$. Genotype BLUEs ${\hat{g}}_{i}$ from this model were then used as the response in GWAS.

Association testing followed the BLINK iterative fixed‐effect modeling framework (M. Huang et al., 2018). Let $y_{i}$ denote the response for individual i (raw phenotypic value or genotype BLUE, depending on the trait). Let $c_{i}$ denote the vector of fixed covariates for individual i, including the intercept, the centered genomic relationship matrix–based correction, and the first three PCs. Let $x_{i}^{\left(Q\right)}$ be the vector of genotypes at the current set of k pseudo–quantitative trait nucleotides (pseudo‐QTNs), $Q=\{q_{1},\text{…},q_{k}\}$, and let $x_{ij}$ denote the genotype of individual i at marker j.

At each iteration, BLINK tests marker j using the following fixed‐effect model, conditional on the current pseudo‐QTN set:

(3) $$y_{i}=c_{i}\alpha +x_{i}^{\left(Q\right)}b+x_{ij}d_{j}+\varepsilon_{i},$$

where α represents the effects of the covariates, b the effects of the pseudo‐QTNs, $d_{j}$ the effect of the tested marker, and $\varepsilon_{i}$ the residual error. This model yields a marker‐specific p‐value.

To update the pseudo‐QTN set, BLINK fits a second fixed‐effect model containing only the covariates and candidate pseudo‐QTNs,

(4) $$y_{i}=c_{i}\alpha +x_{i}^{\left(Q\right)}b+\varepsilon_{i},$$

and selects the optimal subset of pseudo‐QTNs by minimizing the Bayesian information criterion,

(5) BIC=−2LL+kln(n),

where LL is the log‐likelihood of model (4), k is the number of retained pseudo‐QTNs, and n is the sample size.

Between iterations, candidate pseudo‐QTNs are ranked by significance from the testing step, markers failing a Bonferroni‐based screening threshold (α=0.01) are discarded, and markers highly correlated with previously selected pseudo‐QTNs are removed using genotype correlation as a proxy for linkage disequilibrium (LD) (Pearson's r>0.7), following the BLINK algorithm (M. Huang et al., 2018). The testing and selection steps are iterated until the pseudo‐QTN set remains unchanged between successive iterations, indicating convergence.

### 2.4 GS assay

We performed 20 replicates of an 80/20 train–test split for each trait. We conducted GWAS in the training set as above. We then fitted two genomic prediction models, GBLUP and BayesB, under two scenarios: (i) without GWAS hits (baseline), and (ii) with significant GWAS hits fitted as fixed effects. We quantified prediction accuracy in both as the Pearson correlation between predicted genetic values for test lines and their adjusted phenotypes.

We fitted GBLUP with sommer (Giovanny, 2016) as

(6) $$y=\left[\mu +X\;\mathrm{snps}\right]+Z_{g}\;g+\left[Z_{y(l)}\;y\left(l\right)\right]+\varepsilon ,$$

where y is the phenotype vector, μ is the intercept, snps are fixed effects for significant GWAS hits (included only in scenario ii), $g∼N(0,G\sigma_{g}^{2})$ is the random additive genetic effect with G the VanRaden genomic relationship matrix (VanRaden, 2008), $y\left(l\right)∼N\left(0,I\sigma_{y(l)}^{2}\right)$ is the random effect of location nested within year (omitted for traits measured in a single location–year, i.e., mixing energy and $R_{\max}$), and $\varepsilon ∼N(0,I\sigma_{\varepsilon }^{2})$. We estimated variance components by restricted maximum likelihood (REML). When we included GWAS hits as fixed effects, we excluded those markers from G.

We implemented BayesB in BGLR (Pérez & de los Campos, 2014), assigning marker effects $\beta_{j}$ a spike‐and‐slab prior,

$$\beta_{j}∼\left\{\begin{array}{cc} 0, & \text{with probability}\;\pi ,\\ N(0,\sigma_{\beta_{j}}^{2}), & \text{with probability}\;1-\pi , \end{array}\right.$$

with $\sigma_{\beta_{j}}^{2}∼\chi^{-2}\left(\nu ,S^{2}\right)$ and default hyperparameters from BGLR (flat, weakly informative priors so that key hyperparameters are primarily learned from the data, rather than being strongly constrained a priori). We ran chains for 25,000 iterations and discarded the first 5000 as burn‐in. We assessed MCMC mixing and convergence by inspecting trace plots of key posterior summaries (e.g., marker‐variance components and overall genetic value scale) and confirmed that posterior means were stable across iterations and consistent across independent runs with different random seeds. When we modeled GWAS hits as fixed effects, we excluded them from the random‐effect marker set.

### 2.5 Candidate gene identification and functional annotation

We treated Bonferroni‐significant SNPs as candidate loci. For each lead SNP, we retrieved genes within ±50 kb from the *Triticum aestivum* reference genome (IWGSC_RefSeq v1.0; RefSeq Annotation v1.1; Ensembl Plants release 59 GFF). When fewer than three genes fell within this window, we expanded it symmetrically until we identified at least three genes. We queried candidate genes against UniProt (Consortium, 2022) via the application programming interface to identify protein products and putative functions. We contextualized functions using GO terms (Ashburner et al., 2000; Consortium et al., 2023) and cross‐checked annotations with biomaRt (Durinck et al., 2005, 2009).

### 2.6 LD calculations

We quantified LD as $r^{2}$ between all SNP pairs using PLINK 1.9 (Chang et al., 2015; Purcell & Chang, 2015) with parameters –r2 –ld‐window 1000 –ld‐window‐kb 851935 –ld‐window‐r2 0 to include all SNPs. We used LD decay summaries to guide candidate‐gene window sizes.

## 3 RESULTS

### 3.1 Phenotypic variation, trait correlation, PCs, and LD analysis

**FIGURE 1:**
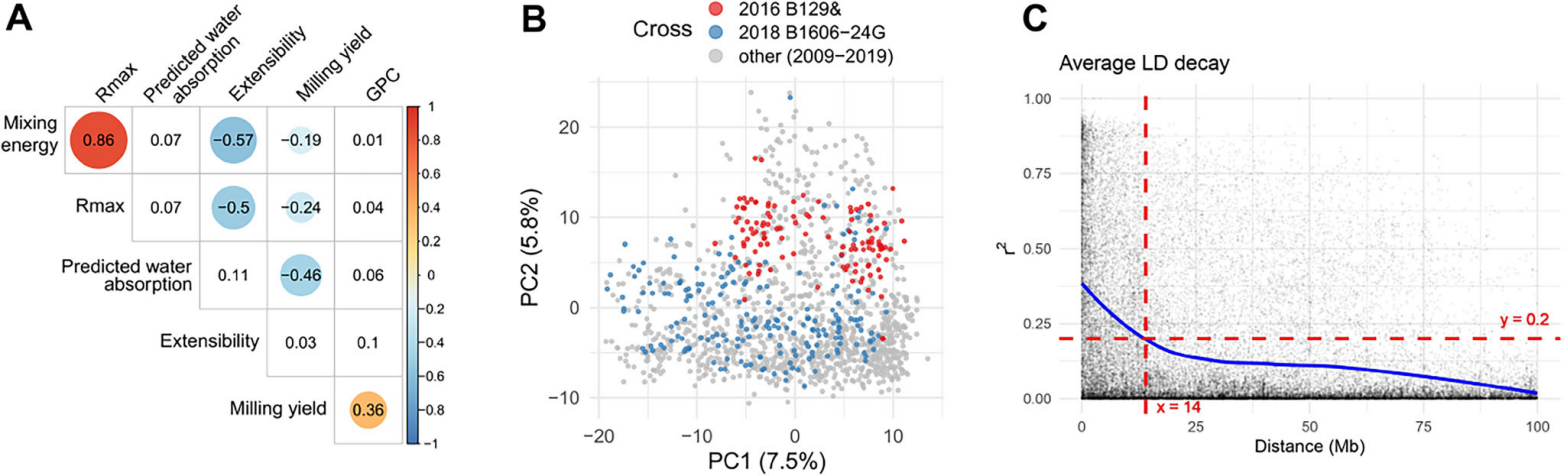
Trait correlations, population structure, and genome‐wide linkage disequilibrium (LD) decay. (A) Pearson correlations among end‐use quality traits. Circle size encodes |r| and color encodes sign; filled symbols denote p<0.05 (two‐sided test). (B) Principal component analysis (PCA) of genotypes (PC1: 7.5%, PC2: 5.8% of variance). Points in red and blue correspond to biparental crosses from 2016 and 2018. Extensibility, milling yield, and water absorption were measured in the 2016 cross (red), whereas all traits except grain protein content (GPC) were measured in the 2018 cross (blue). Gray points denote lines phenotyped only for GPC between 2009 and 2019. (C) LD decay across the genome: each point is pairwise $r^{2}$ versus physical distance; the LOESS (Locally Estimated Scatterplot Smoothing) curve (blue) summarizes the mean trend. Genome‐ wide mean $r^{2}$ drops below 0.2 at ∼14 Mb (cf. 11–30 Mb reported for bread wheat). A similar LD decay plot for individual chromosomes is presented in Figure S2.

We first examined phenotypic and genetic patterns (Figure 1). Stretching energy ($R_{\max}$) correlated strongly and positively with mixing energy, and both traits correlated negatively with extensibility. Milling yield showed negative correlations with both mixing and stretching energies, with the strongest negative association observed with predicted water absorption; in contrast, milling yield correlated positively with GPC.

We assessed population structure with PC analysis. The first two PCs explained 13.3% of the genetic variance (PC1: 7.5%; PC2: 5.8%), indicating minimal stratification overall (Figure 1B). PC2 captured modest separation between the 2016 and 2018 biparental cohorts relative to the broader panel phenotyped primarily for GPC.

Genome‐wide LD decayed to $r^{2}<0.2$ at ∼14 Mb (Figure 1C), consistent with the 11–30 Mb range reported for bread wheat (Chao et al., 2010; Roncallo et al., 2021). Chromosome‐level LD profiles followed a similar trajectory (Figure S2).

### 3.2 GWAS results

**FIGURE 2:**
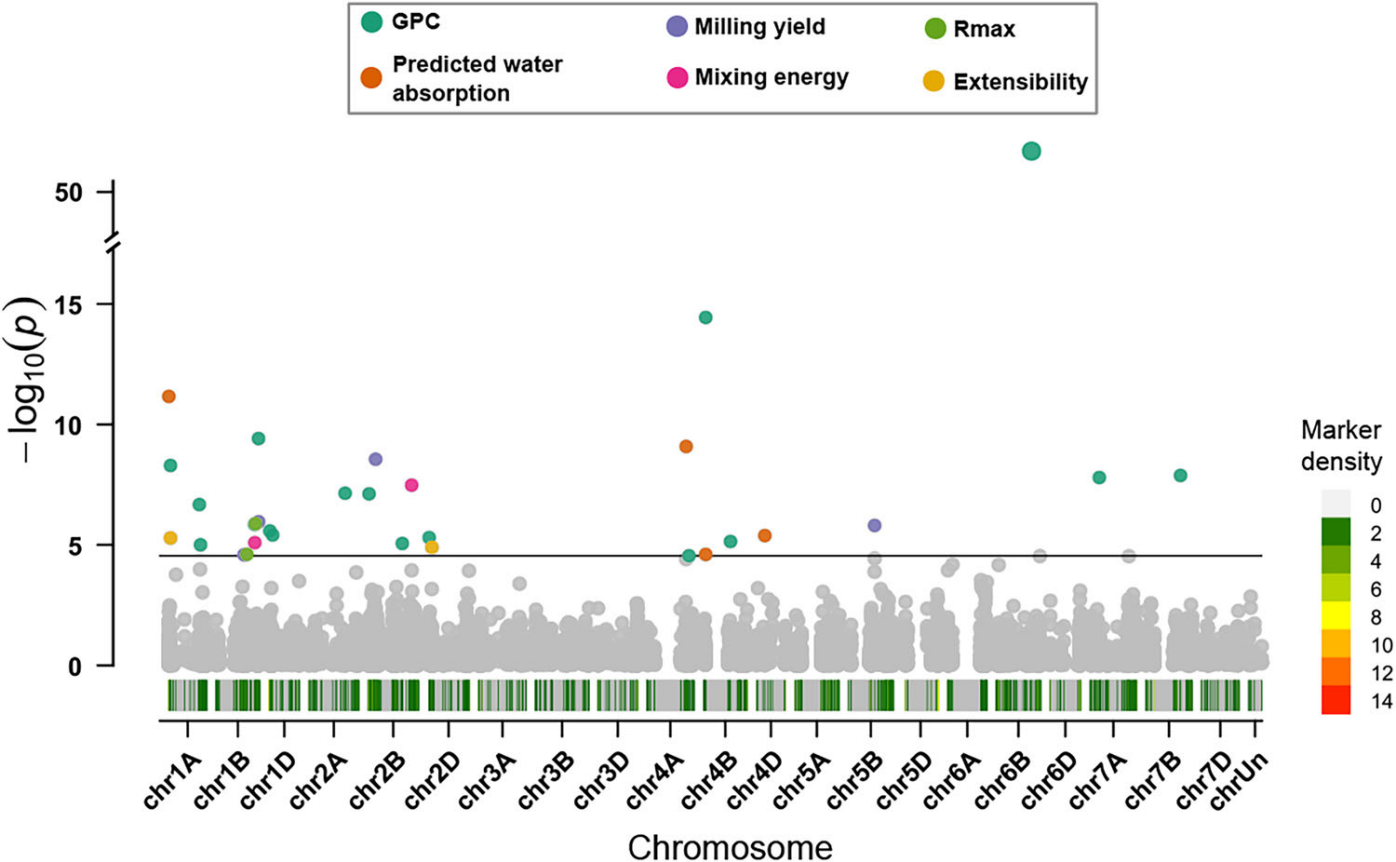
Genome‐wide association signals across six end‐use quality traits. Each point represents a single nucleotide polymorphism (SNP) plotted by genomic position (*x*‐axis) and $-\log_{10}p$ (*y*‐axis). The horizontal line marks the Bonferroni threshold (α=0.05/1767; logarithm of the odds [LOD] =4.55). Points above the threshold are colored by associated trait. The background gradient along the *x*‐axis depicts marker density (gray: none; green: low; red: high). A *y*‐axis break highlights an outlying peak without compressing the remaining scale. See Section 2 for genome‐wide association study (GWAS) model specification.

We detected 29 genome‐wide significant associations at the Bonferroni threshold (α=0.05/1,767; LOD >4.55, where LOD is logarithm of the odds). Two lead SNPs exhibited pleiotropy (i.e., cross‐trait associations with two traits each), yielding 31 distinct marker–trait associations (MTAs) distributed across 13 chromosomes (1A, 1B, 1D, 2A, 2B, 2D, 4A, 4B, 4D, 5B, 6B, 7A, 7B; Table 3; Figure 2). It is worth noting that, although genome‐wide significance was defined using the Bonferroni threshold, Benjamini–Hochberg adjusted p‐values (Benjamini & Hochberg, 1995) are reported to facilitate comparison and ranking of associations under false discovery rate control.

**TABLE 3:** Significant marker–trait associations (MTAs) detected by genome‐wide association study (GWAS).

Trait	SNP	Chrom	Position (bp)	MAF	nobs	Effect	*p*‐value	B.H p‐value	LOD	Maj_all	Min_all	Genes	Dist
Extensibility	RAC875_c16820_419	chr1A	27,603,266	0.25	310	−0.42	5.17E‐06	9.13E‐03	5.29	A	G	TraesCS1A02G046300, TraesCS1A02G046100, TraesCS1A02G046000	149,477, 239,934, 275,270
Extensibility	wsnp_Ex_c6400_11123059	chr2D	65,601,481	0.29	310	−0.38	1.26E‐05	1.11E‐02	4.9	C	T	TraesCS2D02G116500, TraesCS2D02G116600, TraesCS2D02G116400	32,626, 36,518, 64,313
Milling yield	Tdurum_contig48171_189	chr1B	445,021,239	0.24	310	−0.3	2.57E‐05	9.19E‐04	4.59	G	A	TraesCS1B02G252300, TraesCS1B02G252200, TraesCS1B02G252400	84,023, 155,849, 157,079
Milling yield	Tdurum_contig4904_2923	chr1B	674,537,343	0.25	310	−0.89	1.10E‐06	4.92E‐06	5.96	T	C	TraesCS1B02G461500, TraesCS1B02G461600, TraesCS1B02G461700	42,136, 36,751, 36,034
Milling yield	Tdurum_contig77602_931	chr2B	119,853,739	0.05	310	−0.32	2.78E‐09	9.19E‐04	8.56	T	C	TraesCS2B02G152700, TraesCS2B02G152600, TraesCS2B02G152500	219,579, 609,375, 669,031
Milling yield	IAAV5873	chr5B	413,547,967	0.15	310	0.22	1.56E‐06	1.13E‐02	5.81	A	G	TraesCS5B02G234800, TraesCS5B02G234900, TraesCS5B02G234700	61,214, 61,997, 857,440
Mixing energy	Tdurum_contig96049_200	chr1B	613,632,313	0.42	195	0.64	8.08E‐06	7.14E‐03	5.09	C	A	TraesCS1B02G381100, TraesCS1B02G381000, TraesCS1B02G380800, TraesCS1B02G380900	94,592, 206,989, 216,152, 212,728
Mixing energy	wsnp_Ra_c10658_17500498	chr2B	685,900,594	0.12	195	1.6	3.31E‐08	5.84E‐05	7.48	C	T	TraesCS2B02G488500, TraesCS2B02G488600, TraesCS2B02G488700	140,469, 145,038, 148,774
Predicted water absorption	Kukri_c2408_857	chr1A	1,360,891	0.44	310	1.19	6.87E‐12	7.26E‐07	11.16	A	G	TraesCS1A02G002700, TraesCS1A02G002500, TraesCS1A02G002400	12,565, 122,422, 131,552
Predicted water absorption	Excalibur_c2618_1412	chr4A	621,666,044	0.4	310	0.95	8.22E‐10	1.21E‐08	9.09	A	T	TraesCS4A02G340600, TraesCS4A02G340700, TraesCS4A02G340800	1262, 1226, 17,676
Predicted water absorption	Tdurum_contig63670_287	chr4B	38,005,599	0.24	310	0.66	2.48E‐05	1.04E‐02	4.61	T	C	TraesCS4B02G049600, TraesCS4B02G049700, TraesCS4B02G049500	0, 19,842, 135,902
Predicted water absorption	wsnp_BE444858D_Ta_1_1	chr4D	134,619,728	0.12	310	0.83	4.10E‐06	2.42E‐03	5.39	A	G	TraesCS4D02G145700, TraesCS4D02G145600, TraesCS4D02G145500, TraesCS4D02G145800	15,642, 55,602, 69,272, 66,766
Grain protein content	RAC875_c16820_419	chr1A	27,603,266	0.1	1454	0.17	5.06E‐09	2.23E‐06	8.3	A	G	TraesCS1A02G046300, TraesCS1A02G046100, TraesCS1A02G046000	149,477, 239,934, 275,270
Grain protein content	BS00023101_51	chr1A	480,068,381	0.26	1454	−0.16	2.12E‐07	4.16E‐05	6.67	T	C	TraesCS1A02G281400, TraesCS1A02G281300, TraesCS1A02G281200, TraesCS1A02G281600	228,173, 248,295, 354,900, 356,200
Grain protein content	BobWhite_c37384_208	chr1A	498,821,176	0.3	1454	0.11	9.91E‐06	1.09E‐03	5	C	T	TraesCS1A02G307100, TraesCS1A02G307200, TraesCS1A02G307300, TraesCS1A02G307400	24,455, 15,023, 12,034, 9560
Grain protein content	BS00064162_51	chr1B	611,145,616	0.08	1454	0.16	1.40E‐06	2.47E‐04	5.85	A	G	TraesCS1B02G378700, TraesCS1B02G378600, TraesCS1B02G378500	13,444, 179,629, 252,042
Grain protein content	IACX2234	chr1B	671,612,273	0.11	1454	−0.18	3.86E‐10	2.27E‐07	9.41	G	A	TraesCS1B02G457300, TraesCS1B02G457200, TraesCS1B02G457100, TraesCS1B02G457400	40,117, 106,306, 219,037, 219,037
Grain protein content	RAC875_c7752_549	chr1D	13,912,290	0.09	1454	0.15	2.64E‐06	4.25E‐04	5.58	C	T	TraesCS1D02G033900, TraesCS1D02G033800, TraesCS1D02G033700	59,049, 94,627, 109,713
Grain protein content	Excalibur_c5958_1398	chr1D	58,111,434	0.07	1454	−0.15	3.88E‐06	5.72E‐04	5.41	G	A	TraesCS1D02G075700, TraesCS1D02G075800, TraesCS1D02G075900	150,376, 332,774, 337,542
Grain protein content	wsnp_BE403597A_Ta_2_1	chr2A	573,136,296	0.36	1454	0.11	7.17E‐08	1.68E‐05	7.14	A	G	TraesCS2A02G339200, TraesCS2A02G339300, TraesCS2A02G339400	199,591, 230,687, 231,953
Grain protein content	RAC875_c2300_1021	chr2B	17,275,697	0.49	1454	−0.1	7.61E‐08	1.68E‐05	7.12	A	G	TraesCS2B02G036200, TraesCS2B02G036300, TraesCS2B02G036100	35,654, 67,267, 72,638
Grain protein content	Kukri_c25281_99	chr2B	539,255,935	0.11	1454	0.13	8.71E‐06	1.03E‐03	5.06	T	C	TraesCS2B02G376100, TraesCS2B02G376200, TraesCS2B02G376300	21,052, 31,536, 38,481
Grain protein content	Kukri_rep_c113120_104	chr2D	22,008,879	0.08	1454	−0.17	4.97E‐06	6.76E‐04	5.3	A	G	TraesCS2D02G054700, TraesCS2D02G054800, TraesCS2D02G054900	88,246, 100,499, 110,435
Grain protein content	wsnp_Ex_c3988_7221220	chr4A	666,371,209	0.05	1454	−0.16	2.82E‐05	2.93E‐03	4.55	C	T	TraesCS4A02G388700, TraesCS4A02G388900, TraesCS4A02G388600	111,203, 167,416, 219,528
Grain protein content	Tdurum_contig63670_287	chr4B	38,005,599	0.13	1454	0.24	3.65E‐15	3.22E‐12	14.44	T	C	TraesCS4B02G049600, TraesCS4B02G049700, TraesCS4B02G049500	0, 19,842, 135,902
Grain protein content	BS00064884_51	chr4B	426,786,962	0.3	1454	0.1	7.20E‐06	9.09E‐04	5.14	A	G	TraesCS4B02G197800, TraesCS4B02G197900, TraesCS4B02G198000	17,243, 11,516, 279,993
Grain protein content	wsnp_JD_c34368_25929293	chr6B	553,778,886	0.28	1454	0.32	2.77E‐54	4.90E‐51	53.56	C	A	TraesCS6B02G309300, TraesCS6B02G309200, TraesCS6B02G309400	343,676, 362,575, 385,087
Grain protein content	Tdurum_contig13080_153	chr7A	149,047,446	0.41	1454	−0.11	1.60E‐08	4.70E‐06	7.8	T	C	TraesCS7A02G191100, TraesCS7A02G191200, TraesCS7A02G191000	58,991, 119,383, 214,727
Grain protein content	Kukri_c34355_722	chr7B	543,184,224	0.06	1454	0.2	1.31E‐08	4.63E‐06	7.88	G	A	TraesCS7B02G303500, TraesCS7B02G303600, TraesCS7B02G303700	2679, 925,256, 938,999
$R_{\max}$	BS00067146_51	chr1B	488,628,573	0.15	195	46.46	2.46E‐05	2.32E‐03	4.61	T	C	TraesCS1B02G280400, TraesCS1B02G280100, TraesCS1B02G280200, TraesCS1B02G280300	13,893, 145,720, 154,181, 153,297
$R_{\max}$	BobWhite_c48071_144	chr1B	624,345,061	0.35	195	44.77	1.31E‐06	2.18E‐02	5.88	T	C	TraesCS1B02G391100, TraesCS1B02G391000, TraesCS1B02G390900	153,603, 254,937, 261,952

*Note* : For each MTA, we report the SNP identifier, chromosome, and physical position (IWGSC RefSeq v1.0), minor allele frequency (MAF), number of observations (nobs), additive effect (per minor‐allele dosage), nominal p‐value, Benjamini–Hochberg (BH) adjusted p‐value (Benjamini & Hochberg, 1995), logarithm of the odds (LODs) ($-\log_{10}p$), major (Maj_all) and minor (Min_all) alleles, nearby candidate genes, and the physical distance (Dist; bp) from the lead single nucleotide polymorphism (SNP) to each candidate. Only associations surpassing the Bonferroni threshold are shown; full annotations appear in Table S1.

GPC accounted for most signals (17 MTAs), including the strongest association on 6B (LOD =53.56). We identified four MTAs for predicted water absorption (1A, 4A, 4B, 4D) and four for milling yield (1B × 2, 2B, 5B). Extensibility (1A, 2D), mixing energy (1B, 2B), and $R_{\max}$ (1B × 2) each contributed two MTAs.

**FIGURE 3:**
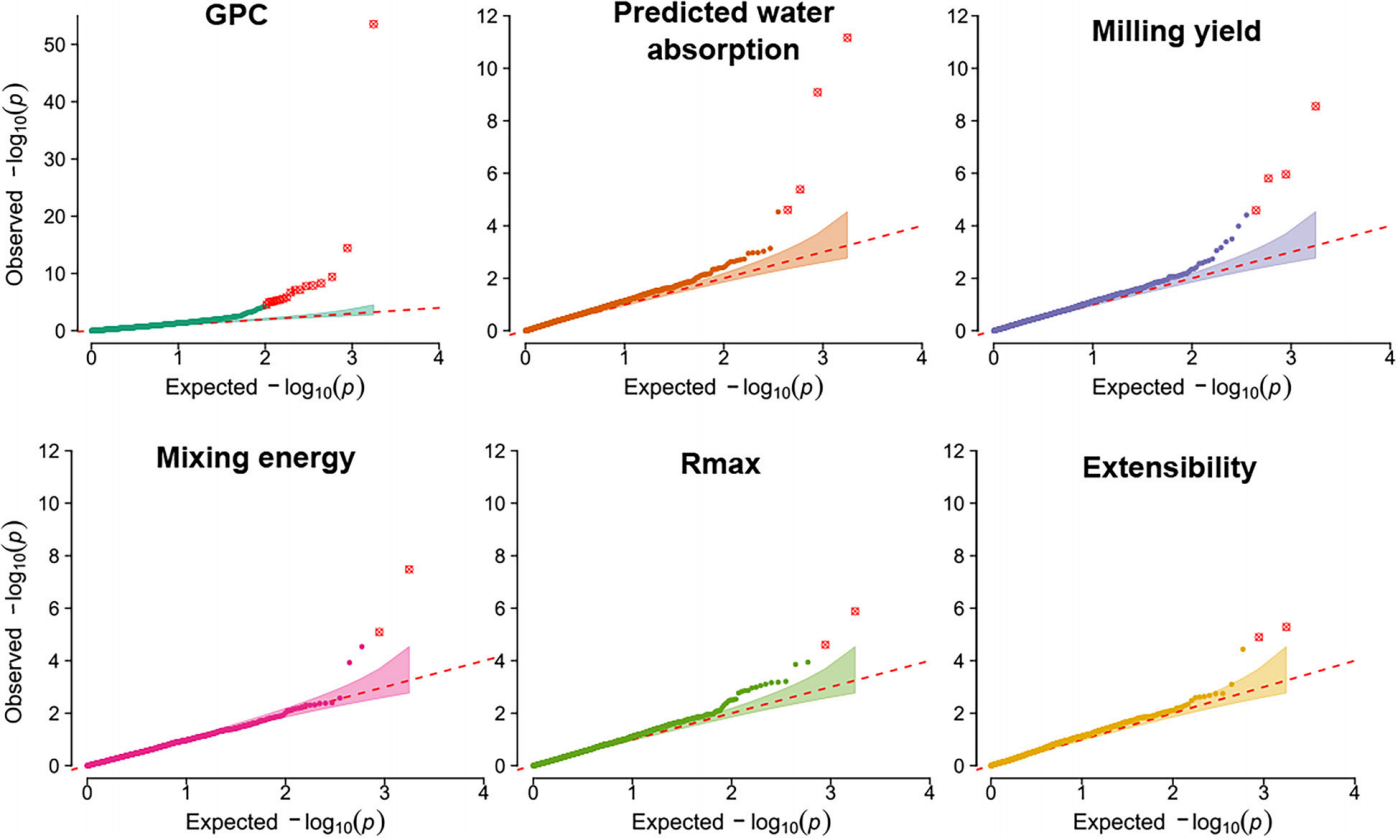
Quantile–quantile (*Q*–*Q*) plots for six traits. Observed $-\log_{10}p$ values (*y*‐axis) are plotted against the null expectation (*x*‐axis). Deviations from the diagonal indicate inflation due to true signal or residual confounding. Symbols in red mark single nucleotide polymorphisms (SNPs) exceeding the Bonferroni threshold (logarithm of the odds [LOD] >4.55). The grain protein content (GPC) panel uses an expanded *y*‐axis to accommodate stronger signals.

We observed two cross‐trait overlaps. On 1A, one SNP associated with both GPC (LOD =8.30) and extensibility (LOD =5.29); on 4B, another SNP associated with GPC (LOD =14.44) and predicted water absorption (LOD =4.61). *Q*–*Q* plots showed trait‐specific departures from the null consistent with these signals and no evidence of broad inflation (Figure 3).

**FIGURE 4:**
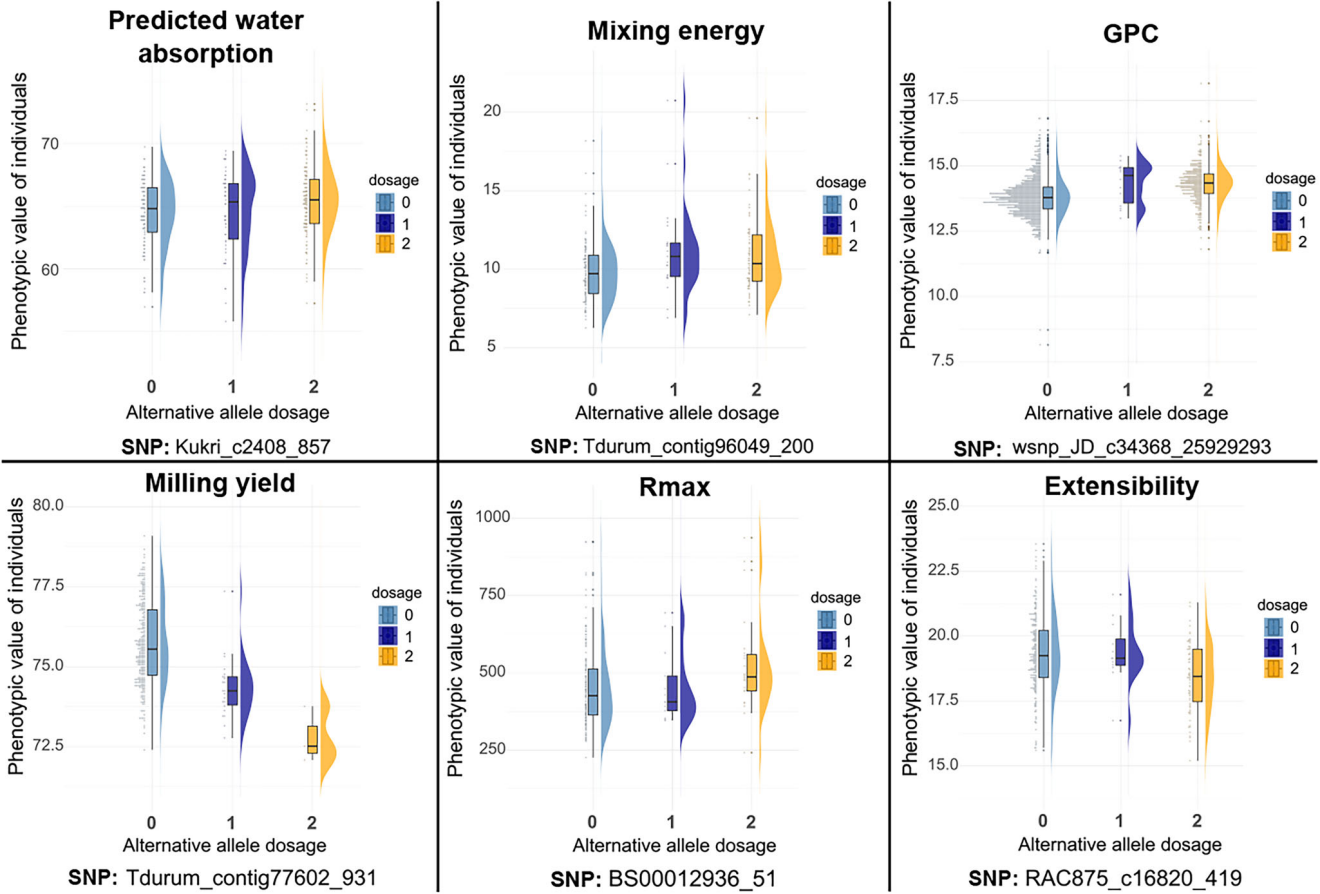
Phenotypic distributions by genotype dosage at the top single nucleotide polymorphism (SNP) for each trait. For the most significant SNP per trait, raincloud plots show the distribution for dosages 0, 1, and 2: density (right), jittered points (left), and a central box‐and‐whisker summarizing the median and interquartile range. Colors distinguish dosages. Units: grain protein content (GPC) (%), milling yield (%), predicted water absorption (%), extensibility (cm), mixing energy (W · h kg^−1^), and $R_{\max}$ (BU).

We quantified phenotypic separation across genotype classes at the top SNP for each trait (Figure 4) to visualize effect direction and magnitude, confirm adequate genotype‐class representation, and check that the association is not driven by sparse classes or outliers. Milling yield displayed the largest genotype‐dependent shift in central tendency and spread, consistent with multiple MTAs of moderate effect. GPC showed clear but tighter separations, in line with its larger sample size and the presence of both very strong (6B) and moderate signals. For mixing energy, $R_{\max}$, extensibility, and predicted water absorption, distributions by dosage were consistent with the smaller number of detected loci.

### 3.3 GS results

**FIGURE 5:**
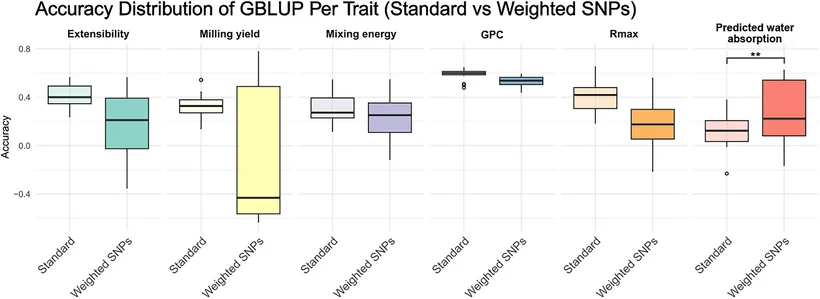
Prediction accuracies with and without fixed genome‐wide association study (GWAS) hits in GBLUP. Boxplots summarize 20 replicates of 80/20 train–test splits for each trait. “Standard” denotes the baseline GBLUP; “Weighted SNPs” includes Bonferroni‐significant GWAS hits as fixed effects (and excludes them from G). Asterisks denote a significant mean increase for predicted water absorption (one‐sided t‐test, p=0.013); other traits show no improvement. Accuracy is Pearson correlation between predictions and adjusted phenotypes in the test set. Same results with the BayesB model are available in Figure S3

We evaluated whether fixing GWAS hits improves prediction. Across 20 replicates of 80/20 train–test splits, including significant SNPs as fixed effects in GBLUP did not increase accuracy for most traits; point estimates were similar or slightly lower than the baseline model (Figure 5). Predicted water absorption was the sole exception, where fixing hits increased mean accuracy 2.24‐fold (one‐sided t‐test, p=0.013). BayesB delivered accuracies comparable to GBLUP across traits (Table 4); the nominal trend toward higher accuracy for predicted water absorption under BayesB was not statistically significant in the standard (no fixed hits) models (p=0.098) and mirrored the GBLUP pattern when fixing hits.

**TABLE 4:** Comparison of mean genomic prediction accuracies between GBLUP and BayesB.

Trait	Accuracy GBLUP	Accuracy BayesB
Extensibility	0.409	0.382
Milling yield	0.332	0.331
Mixing energy	0.308	0.306
GPC	0.590	0.601
$R_{\max}$	0.406	0.408
Predicted water absorption	0.126	0.185*

*Note*: Entries report mean Pearson correlations across 20 replicates of standard models (no fixed genome‐wide association study [GWAS] hits). The asterisk indicates a nominal one‐sided t‐test p=0.098 for the difference between models in predicted water absorption; differences for other traits are negligible. See Section 2 for cross‐validation design and BayesB hyperparameters.

### 3.4 Candidate genes and functional context

**FIGURE 6:**
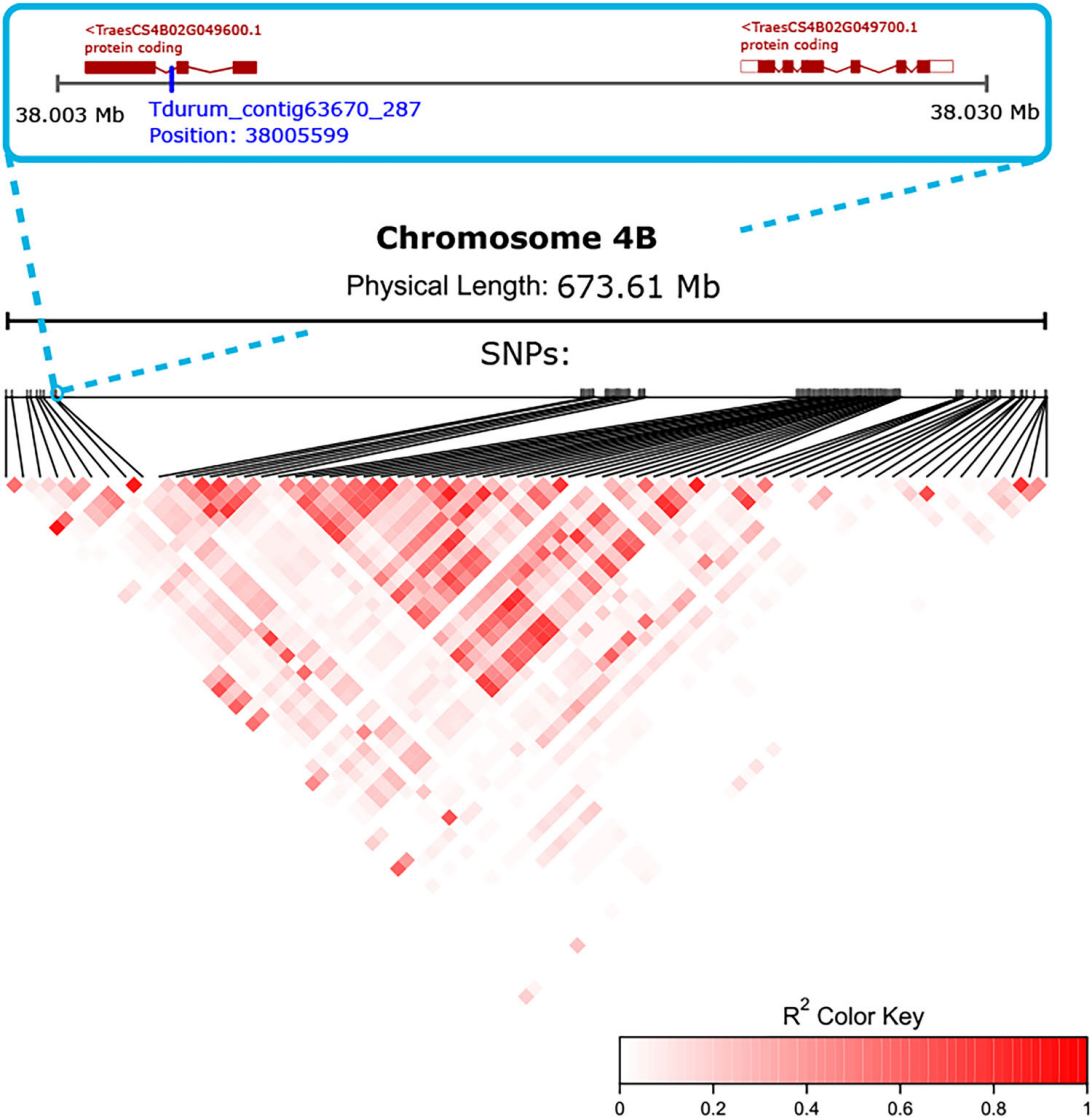
Local linkage disequilibrium (LD) architecture around a lead single nucleotide polymorphism (SNP) on chromosome 4B. Bottom: pairwise LD ($r^{2}$) among SNPs rendered as a heat map (red: higher $r^{2}$). Middle: genomic coordinates along 4B with SNP positions. Top: zoom‐in on the lead SNP (blue) that associates with both grain protein content (GPC) and predicted water absorption, with nearby candidate genes highlighted. Coordinates refer to IWGSC RefSeq v1.0. See Section 2 for LD computation and candidate‐gene selection.

We considered as candidate genes those located within ±50 kb of each lead SNP (and, when fewer than three genes were captured, expanding the search window until at least three genes were included) and summarized putative functions via UniProt and GO terms (Table 3; Table S1). All SNP–gene distances were far below the empirical LD‐decay scale (∼14 Mb); the largest distance was 939 kb. Figure 6 shows the LD pattern across the entire chromosome 4B, providing a chromosome‐wide view of LD in relation to the pleiotropic association detected on this chromosome. On chromosome 4B, the lead SNP associated with both GPC and predicted water absorption is located within an intron, and its position is shown in Figure 6 relative to two of the closest candidate genes considered in the UniProt‐based annotation. This representation illustrates how the significant marker relates spatially to nearby annotated genes within the context of LD along the full chromosome. The well‐known *Rht‐B1* locus lies ∼8 Mb away and remains of contextual interest given extended LD in wheat.

For predicted water absorption, candidates on 1A implicate histone modification, phospholipid transport, and electron‐transport chain components, whereas signals on 4A/4B/4D point to protein folding, trafficking, and stress‐response pathways. Mixing energy candidates on 1B and 2B include ribosomal constituents and transcriptional regulators. For milling yield, two 1B signals neighbor genes with RNA‐polymerase binding and receptor kinase–like domains; the 2B signal implicates phosphatidylinositol biosynthesis and proteolysis‐related transmembrane proteins; the 5B signal highlights photosynthetic electron transport (photosystem I iron–sulfur centers and NAD(P)H–quinone oxidoreductase).

For $R_{\max}$, signals on 1B sit near genes encoding DUF1618 family proteins and transmembrane proteins involved in peptide transport. Extensibility candidates include 1A genes with carbohydrate‐hydrolase activity and 2D genes encoding proteins with RING/zinc‐finger domains and esterases.

GPC yielded the largest number of associated SNPs, spanning diverse functions (Table 3; Table S1). On 1A, candidates include genes for δ‐1‐pyrroline‐5‐carboxylate biosynthesis, transcription factors, vesicle‐mediated transport, microtubule binding, ribosomal proteins, calcium‐dependent kinases, endonucleases, and carbohydrate hydrolases (also noted for extensibility). On 1B, one signal maps near poorly characterized genes, and another near genes encoding the H3.2 histone and a histone H4 variant. On 1D, candidates include O‐methyltransferases, chalcone‐biosynthesis enzymes, a KN1‐type homeobox transcription factor, and a fertility‐restorer protein. Across 2A/2B/2D, four signals highlight a large ribosomal‐subunit protein, a PDZ domain–containing protease, and enzymes such as cytochrome P450s and glycosyltransferases. On 4A, candidates encode an α‐carbonic anhydrase and a fatty acyl‐CoA reductase; on 4B, proteins with PCI, CNH, and PHD (plant homeodomain) domains, together with L‐ascorbate peroxidase and a MAP kinase; on 6B, a fatty‐acid desaturase domain protein and a transmembrane transporter; on 7A, a putative transcription factor and a transmembrane water channel; and on 7B, proteins with DYW and F‐box domains plus a gene involved in carbohydrate metabolism.

**FIGURE 7:**
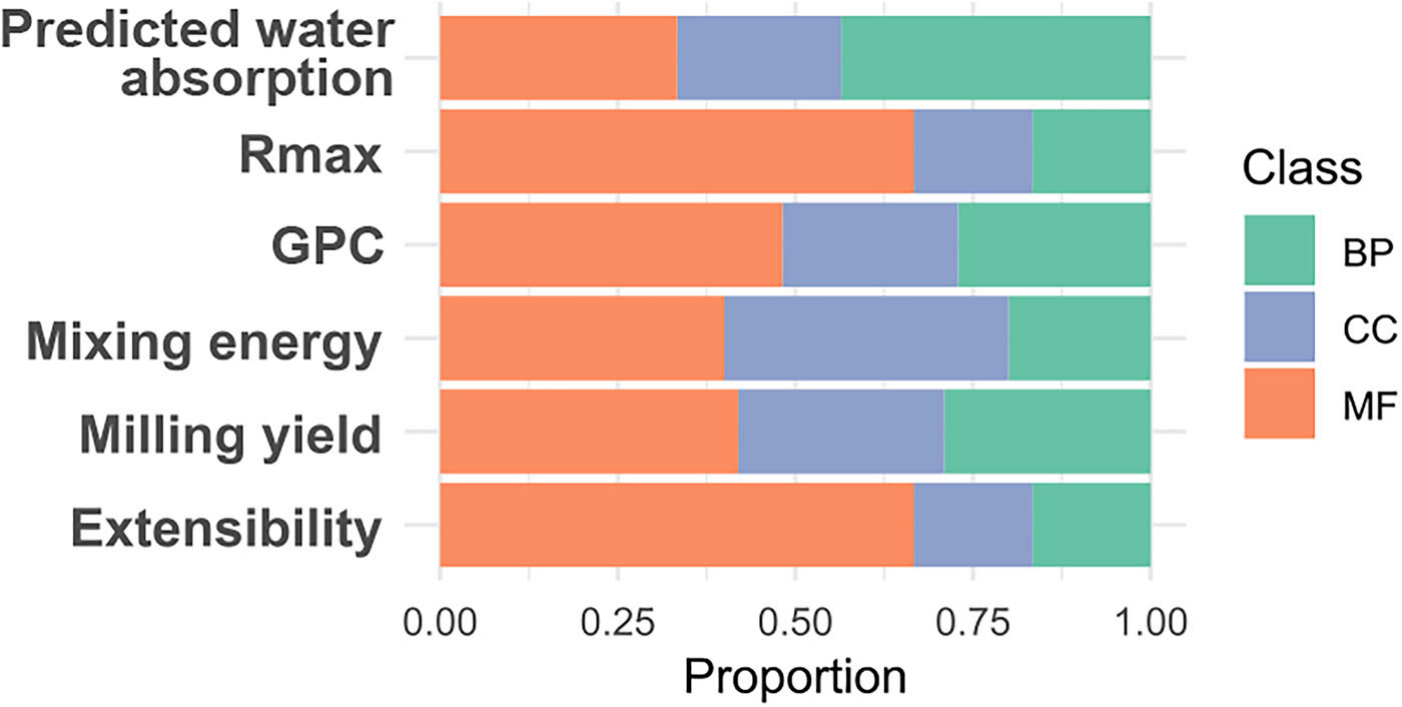
Functional composition of candidate genes across traits. Stacked bars show the proportion of gene ontology (GO) namespaces assigned to candidate genes per trait: biological process (BP), cellular component (CC), and molecular function (MF). Traits $R_{\max}$ and extensibility display similar GO profiles, whereas predicted water absorption differs most from the others. Full GO term lists are provided in Table S1.

Figure 7 summarizes GO class distributions by trait. $R_{\max}$ and extensibility show nearly identical GO profiles, whereas GPC, mixing energy, and milling yield share partly overlapping profiles distinct from those two. Predicted water absorption displays the most divergent GO composition. Full gene‐level annotations, domains, sources, and GO terms appear in Table S1.

## 4 DISCUSSION

### 4.1 Integrating GWAS findings with established loci

We mapped multiple MTAs on chromosome group 1 across all traits, many near glutenin and gliadin loci long implicated in wheat quality (Campbell et al., 2001; D. Liu et al., 2023; Huebner & Wall, 1976; McCartney et al., 2006; Nelson et al., 2006; Sharma et al., 2020; Shewry et al., 2003; Sozinov & Poperelya, 1982; Sun et al., 2008; White et al., 2022; X. Huang et al., 2006; Zanetti et al., 2001; Zhao et al., 2020; Zhou et al., 2021). For GPC, two MTAs on 1A at 480 and 498 Mb lie close to *Glu‐A1* (510 Mb), which encodes HMW‐GS central to dough strength and elasticity (Anjum et al., 2007; Zhang et al., 2018). A shared 1A signal at 27 Mb for extensibility and GPC aligns with an extensibility MTA at ∼29 Mb reported by Zhao et al. (2024). The 1A association at ∼5 Mb for water absorption implicates *Glu‐A3*, *Gli‐A1*, and *Gli‐A3*, consistent with the influence of storage‐protein loci on processing quality (Anjum et al., 2007).

Chromosome 1B harbored the largest number of associations across traits, yet we found no unambiguous correspondence with known glutenin/gliadin loci, pointing to novel QTL or under‐characterized regions affecting milling yield, mixing energy and $R_{\max}$ (Campbell et al., 2001). On 1D, the GPC signal at 14 Mb lies near *Glu‐D3* and *Gli‐D4*, in line with the role of storage proteins in end‐use quality (Zhou et al., 2021); the 58 Mb MTA lacks a clear candidate among established loci.

On group 2 chromosomes, we detected MTAs for GPC (2A, 2B, 2D), milling yield (2B), and mixing energy (2B). The 2B signals at ∼120 Mb (milling yield) and ∼686 Mb (mixing energy) overlap regions previously implicated for GPC (Campbell et al., 2001; J. Liu et al., 2019; Muqaddasi et al., 2020; Nigro et al., 2019; White et al., 2022), whereas other 2A/2D/2B findings appear novel.

A key result was the strong 4B association at 38 Mb shared by GPC and water absorption. Because this region lies near *Rht‐B1*, a gibberellin‐insensitive dwarfing gene with large effects on yield (Flintham et al., 1997; Peng et al., 1999), we propose *Rht‐B1* underlies these QTL. Prior work connects *Rht‐B1* to reduced GPC (Gent & Kiyomoto, 1997; W. Hu et al., 2024), and GPC can modulate water absorption (Rezette et al., 2025), offering a parsimonious explanation for the dual signal.

The strongest signal in our study mapped to 6B for GPC (553 Mb). Although *Gpc‐B1* (*NAM‐B1*; TraesCS6B02G207500LC) is a compelling biological candidate (Bordes et al., 2011; Kumar et al., 2018; White et al., 2022), its reported position (∼134 Mb) is distant (Joppa et al., 1997; Olmos et al., 2003; Uauy et al., 2006). A nearby *NAM‐B1*‐like pseudogene (LOC123134508) may instead explain the association, a hypothesis supported by a recent report of a GPC MTA close to our peak (Zhao et al., 2024). Together, these results reveal candidate regions consistent with known biology and highlight additional, potentially novel QTL on 1B, 1D, 2A, 2B, 2D, 4A, 4B, 4D, 5B, 7A, and 7B that merit validation.

Additionally, we will like to note that haplotype‐based analyses could provide additional resolution for validating associated loci; however, the relatively low genome‐wide marker density in this study limits the reliable definition of local haplotypes or LD blocks at the gene scale. Haplotype‐based validation of these loci remains an important direction for future work using higher density genotyping data.

### 4.2 Impact of incorporating GWAS signals into genomic prediction

We found limited benefit from fixing significant GWAS hits in genomic prediction, indicating predominantly polygenic control for the evaluated quality traits. Across traits, adding a small set of Bonferroni‐significant markers to GBLUP did not improve accuracy and sometimes reduced it (Figure 5). BayesB, which can capitalize on major loci, did not outperform GBLUP (Table 4), reinforcing a highly polygenic architecture (Dong et al., 2016; X. Wang et al., 2019). Despite this, cross‐validated accuracies support GS as a practical tool for these costly‐to‐phenotype traits (Battenfield et al., 2016; Juliana et al., 2019).

Water absorption was the exception: fixing GWAS hits increased accuracy (Figure 5), and BayesB showed a nominal advantage (Table 4). Such a response is consistent with theoretical expectations that incorporating major loci as fixed effects is most effective for traits controlled by a small number of QTL with relatively large effects, rather than by highly polygenic architectures (Bernardo, 2001, 2014). This pattern suggests a larger effect component for water absorption, consistent with known influences of GPC, kernel hardness, and storage proteins (Campbell et al., 2001). In line with this interpretation, the low number of GWAS hits detected for predicted water absorption supports a simpler genetic architecture dominated by a few large‐effect loci, which likely explains why fixed‐effect modeling was beneficial for this trait. Notably, we did not detect associations at *Pina*/*Pinb* for kernel hardness, despite reports elsewhere (Campbell et al., 2001); instead, our analyses pointed to alternative, possibly novel loci (Section 4.1). If validated, such loci could represent promising targets for marker‐assisted selection, further underscoring the practical relevance of this result.

Methodologically, we avoided data leakage by discovering GWAS hits within the training set before incorporating them into prediction models. Studies that select hits on the full dataset and then fix them for GS risk circularity and inflated gains (Spindel et al., 2015, 2016). The lack of widespread improvement here likely reflects this stricter, unbiased workflow and provides a realistic estimate of the value of fixed‐effect incorporation for these traits.

### 4.3 GO‐driven insights into candidate genes

We used gene annotations near lead SNPs to generate testable hypotheses about mechanism. Two milling‐yield MTAs illustrate this approach. On 5B (413 Mb), nearby genes encode NAD(P)H–quinone oxidoreductase subunit 4L and a photosystem I iron–sulfur center protein, key components of photosynthetic electron transport. Enhanced photosynthesis can increase biomass and starch deposition (Essemine et al., 2016; Kang et al., 2013), thereby influencing milling yield (Kim & Kim, 2021); lines carrying this marker differed in yield (Table S2), supporting this link. On 1B (445 Mb), a neighboring aminotransferase‐like, plant mobile domain protein implicated in meristem maintenance may affect amino acid metabolism and, by extension, GPC and grain quality (Barneix, 2007; DePauw et al., 2007; Ühlken et al., 2014); corresponding yield differences were again evident (Table S2).

For mixing energy, the 1B (613 Mb) region lies near a large ribosomal subunit gene (eL28). Ribosomal proteins support translation and participate in stress responses; in rice, eL28‐like genes respond to abiotic stress and show strong expression in vegetative tissues (Moin et al., 2016). Stress can alter GPC and gluten composition (X. Wang et al., 2018), with downstream effects on dough rheology (Kuktaite et al., 2004); our *t*‐tests are consistent with yield and GPC shifts at this locus (Table S2). For water absorption, the 4D (134 Mb) MTA neighbors a small heat‐shock protein gene, aligning with heat‐stress protection (Reddy et al., 2014) and potential impacts on grain development and quality (X. Wang et al., 2018).

These functional interpretations remain provisional. Fine‐mapping, allele mining, and targeted validation (e.g., near‐isogenic lines, gene editing, and expression profiling during grain filling) will be required to establish causality and quantify effects useful for breeding.

## 5 CONCLUSION

We show that GPC and related end‐use quality traits in bread wheat are highly polygenic, with signals distributed across many small‐effect loci. Associations near canonical loci, like*Glu‐1*, *Rht‐B1*, and the *Gpc‐B1*/*NAM‐B1* region, remain central for improvement, while additional MTAs on 4D and 5B expand the set of actionable targets, including for under‐studied rheological traits.

Using an unbiased workflow that involved splitting data into training and test sets, discovering GWAS hits only in the training set, and then fixing those hits in prediction, we observed no systematic accuracy gains and occasional declines. These results indicate that, for polygenic traits, GS derives most of its utility from genome‐wide marker information rather than from a narrow set of significant GWAS hits that explain limited variance. This design provides a realistic estimate of marker utility and underscores the need to avoid data leakage when integrating GWAS with GS.

GO‐based annotation offered plausible biological context for candidate genes but remains provisional. We identify clear next steps: fine‐mapping, functional validation (e.g., targeted editing and expression profiling during grain fill), and higher throughput, standardized phenotyping to resolve effect sizes and mechanisms. By coupling rigorous GWAS practice with cross‐validated GS, breeders can more efficiently exploit both large‐effect QTL where they exist and the diffuse polygenic background to accelerate gains in wheat nutritional and processing quality.

## AUTHOR CONTRIBUTIONS

**Juan Menor de Gaspar**: Data curation; formal analysis; investigation; methodology; writing—original draft; writing—review and editing. **Richard D. Cuthbert**: Funding acquisition; resources; writing—review and editing. **Ruan Yuefeng**: Data curation; funding acquisition; investigation; resources; writing—review and editing. **Ron Knox**: Funding acquisition; resources; supervision; writing—review and editing. **BinXiao Fu**: Methodology; writing—review and editing. **Kun Wang**: Methodology; writing—review and editing. **Jatinder S. Sangha**: writing—review and editing. **Samia Berraies**: writing—review and editing. **Brad Meyer**: Methodology; resources; supervision; writing—review and editing. **Firdissa E. Bokore**: writing—review and editing. **Julio Isidro y Sánchez**: Conceptualization; data curation; funding acquisition; project administration; supervision; validation; writing—original draft; writing—review and editing

## CONFLICT OF INTEREST STATEMENT

The authors declare no conflicts of interest.

## Supporting information



Data S1

Data S1

Data S1

## Data Availability

The datasets analyzed and generated during the current study are available in our GitHub repository at https://github.com/Brad‐Meyer/AAFC‐SCRDC_Publications.
