# Supplementary material for: Genetic control of wheat flour end‐use quality and rheology by genome‐wide association studies
Source: Plant Genome. 2026 Apr 16;19(2):e70236. doi: 10.1002/tpg2.70236 (PMC13087497; doi:10.1002/tpg2.70236)
Supplement: Supplementary file 1 [file TPG2-19-e70236-s001.pdf]

SUPPLEMENTARY MATERIAL

Supplementary Tables S1 and S2 are available for download on the publisher's website.

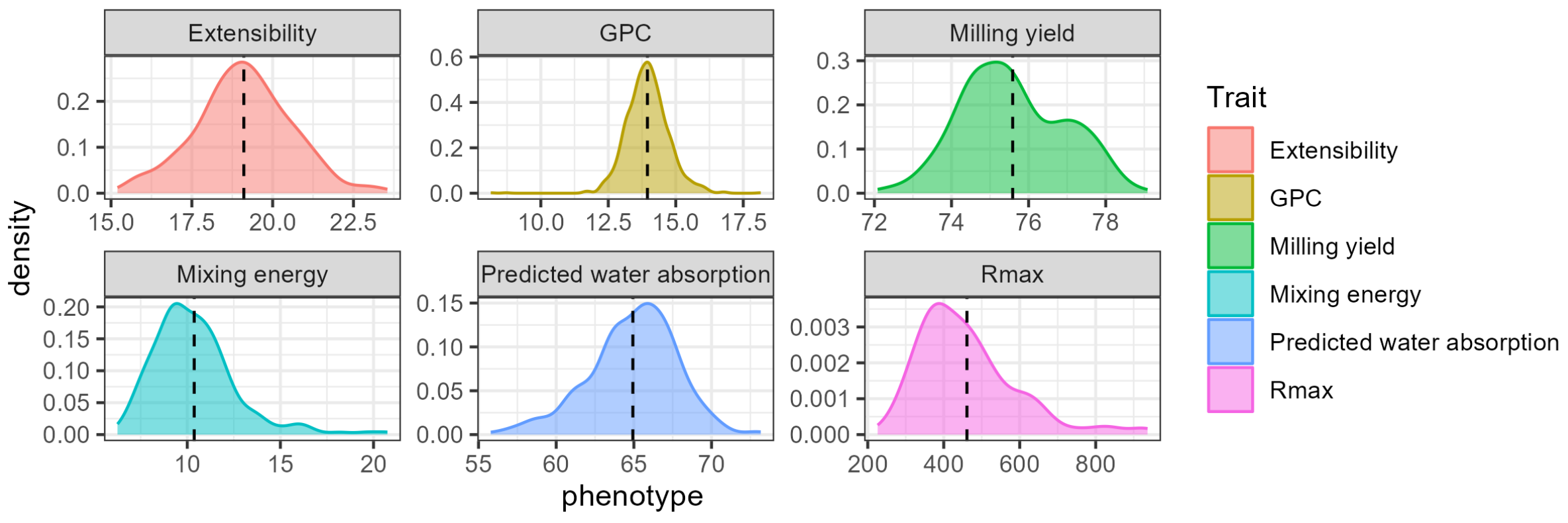

FIGURE S1 Phenotypic distributions of the analyzed traits. All traits are displayed as raw phenotypic measurements, with the exception of GPC, for which Best Linear Unbiased Estimates (BLUEs) are shown. (Generated using *ggplot2*(**ggplot2**))

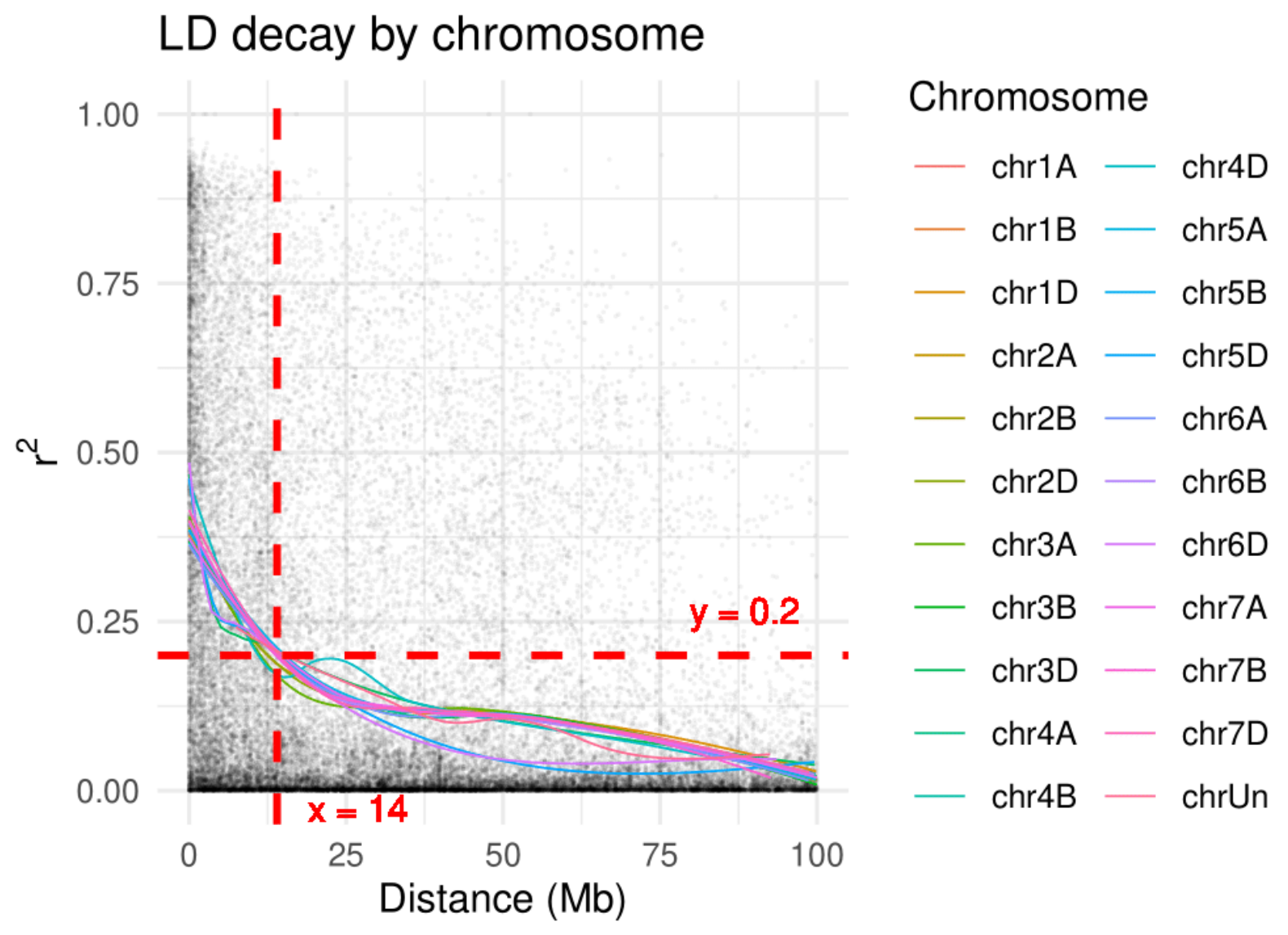

FIGURE S2 Linkage disequilibrium decay per chromosome plot. In this plot each point represents the correlation coefficient r^2 between a pair of SNP's vs. the distance between them. Different colored lines are shown representing the mean r^2 per distance on each chromosome, smoothened with the LOESS method (Generated using *ggplot2*(**ggplot2**))

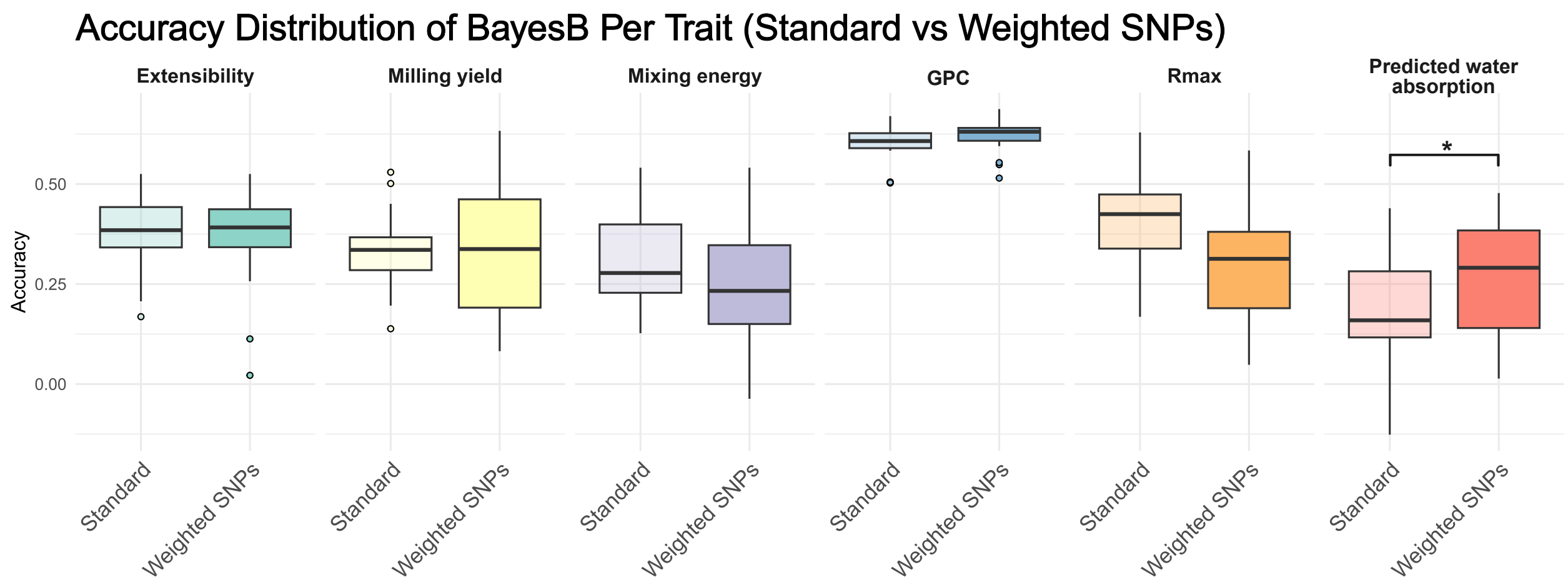

FIGURE S3 **Boxplots of BayesB Model Accuracies** Boxplots comparing the accuracy of the BayesB model when significant GWAS hits are included as fixed effects (Weighted SNPs) versus the standard BayesB model (Standard) (Generated using `ggplot2` (**ggplot2**)). **(*)** Difference in means for water absorption is significant (one-sided t-test, $p = 0.044$).
